# Functional traits shape small mammal-helminth network: patterns and processes in species interactions

**DOI:** 10.1017/S0031182021000640

**Published:** 2021-07

**Authors:** Thiago dos Santos Cardoso, Cecilia Siliansky de Andreazzi, Arnaldo Maldonado Junior, Rosana Gentile

**Affiliations:** 1 Laboratório de Biologia e Parasitologia de Mamíferos Silvestres Reservatórios, Instituto Oswaldo Cruz, Fundação Oswaldo Cruz, Av. Brasil 4365, Manguinhos, 21045-900, Rio de Janeiro, RJ, Brasil; 2 Programa Fiocruz de Fomento à Inovação – INOVA FIOCRUZ, Fundação Oswaldo Cruz, Av. Brasil 4365, Manguinhos, 21045-900, Rio de Janeiro, RJ, Brasil

**Keywords:** Abundance, centrality, functional ecology, marsupials, nematodes, network analysis, neutral theory, niche theory, parasitism, rodents

## Abstract

Understanding the role of species traits in mediating ecological interactions and shaping community structure is a key question in ecology. In this sense, parasite population parameters allow us to estimate the functional importance of traits in shaping the strength of interactions among hosts and parasites in a network. The aim of this study was to survey and analyse the small mammal-helminth network in a forest reserve of the Brazilian Atlantic Forest in order to understand (i) how functional traits (type of parasite life cycle, site of infection in their host, host and parasite body length, host diet, host locomotor habit and host activity period) and abundance influence host–parasite interactions, (ii) whether these traits explain species roles, and (iii) if this relationship is consistent across different parasite population parameters (presence and absence, mean abundance and prevalence). Networks were modular and their structural patterns did not vary among the population parameters. Functional traits and abundance shaped the interactions observed between parasites and hosts. Host species abundance, host diet and locomotor habit affected their centrality and/or vulnerability to parasites. For helminths, infection niche was the main trait determining their central roles in the networks.

## Graphical abstract

**Figure:**
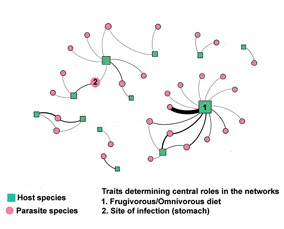

## Introduction

The mechanisms associated with the evolution of parasite's host ranges, i.e. the number of host species in which a parasite occurs, can be understood by the concept of encounter (related to biodiversity and behaviour) and compatibility (related to resource and defence traits) filters (Combes, 2001). These filters are based on the niche theory and mediate host–parasite interactions, as certain host attributes would increase their chance of acquiring parasite infections, and parasite traits would influence their degree of specialization on hosts (Combes, 2001). Ecological traits (e.g. host locomotor habit, diet and activity period, and type of parasite life cycle) are more related to the encounter filter than to the compatibility, while morphological, physiological and immunological traits (e.g. host and parasite body length, and parasite infection site) are related to the compatibility filter (Poulin, 2007). In addition, host–parasite interactions are expected to be proportional to their abundances, which supports that both abundance-driven neutral processes and trait-based niche constraints can drive interaction patterns in host–parasite networks (Runghen *et al*., 2021). Moreover, the number of potential interactions among hosts and parasites can be constrained by phylogeny, which limits the interactions to a subset of species with shared coevolutionary history (Poulin, 2010; Pilosof *et al*., 2015).

Network analysis contributes to parasite ecology studies by allowing the modelling of factors associated with parasite transmission among hosts (Luis *et al*., 2015; Runghen *et al*., 2021) in order to determine the most important ecological processes that structure parasite communities. It also provides information to understand the functional role played by species in a community (Poulin, 2010). In an interaction network, parasite abundance and prevalence rates can be used to describe the strength of interactions between hosts and parasites (Poulin, 2010; Bellay *et al*., 2015). The number, strength and distribution of interactions among species describe the importance of each host and parasite species in the transmission process (Delmas *et al*., 2019; Runghen *et al*., 2021). Network patterns can be related to community dynamics in the sense that central host species may be sources of many parasites for other species, connecting different transmission cycles in the network (Poulin, 2010; Runghen *et al*., 2021). Species importance in the network can be evaluated by using centrality measures (Costa *et al*., 2007). This analysis helps to understand the influence of species traits in explaining the strength of interactions among species. However, it is still a challenge to understand how the functional traits of host and parasite species are related to their structural role in host–parasite networks.

Differences in quantitative patterns of interactions among species reflect the heterogeneity in host vulnerability to parasites and parasite dependence on its hosts (Bellay *et al*., 2015), and may be considered as a measure of mutual dependence between a given host and a parasite species in a network, i.e. the species strength (Bascompte *et al*., 2006). Therefore, the dependence of a parasite species on a given host refers to the number of interactions this parasite has with this host in relation to all the others in the network. In turn, the vulnerability of a host species to a certain parasite refers to the number of interactions this host has with this parasite in relation to all the others (Bellay *et al*., 2015).

Studies on host–parasite interaction networks have helped to elucidate the ecological role that species play on the dynamics of infections in the environment (Luis *et al*., 2015; Bordes *et al*., 2017; Stella *et al*., 2018; Dallas *et al*., 2019; Nieto-Rabiela *et al*., 2019). For instance, species centrality, which represents the importance of a particular species to the structure of the network (Newman, 2010), reflects the vulnerability of a novel host species to acquire parasites and pathogens from reservoir host populations, which is the spillover risk (Bordes *et al*., 2017; Nieto-Rabiela *et al*., 2019).

Recent studies using helminths and other groups of parasites have shown that the mode of transmission of these parasites, as well as age, population density, geographic distribution or host phylogeny, is recurrent underlying drivers of the structure of host and parasite interactions (Dallas *et al*., 2019; Bellay *et al*., 2020; Llopis-Belenguer *et al*., 2020). However, it is still unclear if these drivers equally affect different parameters related to the strength of host–parasite interactions, such as the presence–absence of parasite species, and parasite abundance and prevalence.

Previous studies analysed the helminth metacommunity of sigmodontine rodents (Cardoso *et al*., 2018) and the didelphid marsupial *Didelphis aurita* Wied-Neuwied, 1826 (Costa-Neto *et al*., 2019) in the same study area as the present study using the Elements of Metacommunity Structure analysis (Leibold and Mikkelson, 2002). Later, Cardoso *et al*. (2020) investigated the mechanisms responsible for the diversity of the helminth metacommunity of rodents and marsupials. The authors found that host attributes (host body mass, host diet and helminth species richness), as well as spatial variables at a broad spatial scale (among localities), were the most important factors explaining the variation in helminth species abundance at the infracommunity level, i.e. parasite community within an individual host.

The aim of this study was to investigate whether abundance, functional traits or taxonomic distance can explain the role of species in the interaction network of small mammals (marsupials and rodents) and helminths at Serra dos Órgãos National Park (PARNASO), southeastern Brazil. The importance of functional traits in explaining the role played by host and parasite species in the local network was analysed considering parasite population parameters (presence and absence, mean abundance and prevalence). Species centralities were characterized and host traits were related to their vulnerability to parasite infection, as well as parasite traits to their dependence on hosts. The following hypotheses were tested: (1) Host and parasite functional traits and their abundances influence the number and strength of interactions in the network; (2) Functionally and taxonomically similar host species share more parasites with each other than dissimilar hosts. Likewise, functionally and taxonomically similar parasites exploit host species that are more similar among each other than dissimilar ones.

## Materials and methods

### Study area and data source

This study is part of a research project on Atlantic Forest biodiversity, which investigated the taxonomic, evolutionary, ecological and parasitological aspects of several taxa. The study was carried out at PARNASO, a preserved forested area of 20,024 ha in the state of Rio de Janeiro, internationally recognized as a Biosphere Reserve and one of the most important remnants of Atlantic Forest in Brazil. Data were collected in late spring 2014 (rainy season) and winter 2015 (dry season), in three localities: Bonfim (22°27′36.2″S 43°05′37″W; 1074 m height), Barragem do Caxambú (22°30′20″S 43°06′47.5″W; 1117 m height) and Uricanal (22°29′20.5″S 43°07′27.8″W; 1056 m height). See Cardoso *et al*. (2018) for more details of the study area and sampling methods.

### Functional trait data for host and parasite species

Morphological and ecological traits related to host and parasite life-history were obtained from our database and literature (Supplementary Table S1). Host species traits were: body length, diet, locomotor habit and activity period. Parasite species traits were: body length, site of infection (infection niche) in their host and the type of life cycle. These traits were chosen because they are considered important predictors of host–parasite interactions. Host and parasite body length and site of infection may limit the parasite abundance in infrapopulations, i.e. population of a parasite species within an individual host. Host diet, locomotor habit and activity period may influence host exposure and contact with parasites (Guégan *et al*., 2005; Poulin, 2007; Morand, 2015; Dallas *et al*., 2019).

### Data analysis

A full network including all small mammal species captured during the study and all helminths recovered was analysed in order to describe the host–parasite interaction patterns. Nodes represent host and parasite species and links among nodes represent the observed species interactions. To build the host–parasite interaction network, we used all species of small mammals, including species that were not parasitized by helminths (i.e. no interactions with parasites). We consider that the absence of interactions is also informative for studies of parasite ecology, allowing to investigate the sharing of characteristics between species without interactions. Three parameters were used to infer species strength: (i) presence and absence of observed interactions, (ii) mean abundance of each parasite per host species and (iii) prevalence of each parasite per host species. Helminths mean abundance and prevalence (Supplementary Table S2) were calculated for each parasite species in each host species according to Bush *et al*. (1997). Helminth mean abundance represents the total number of helminth individuals recovered divided by the total number of small mammal individuals examined (Bush *et al*., 1997). The prevalence represents the proportion of the infected hosts for a given helminth species in relation to the total number of small mammal individuals examined (Bush *et al*., 1997). The network representation was built using the software Gephi 0.9.2 (Bastian *et al*., 2009).

All the following analyses were performed in the software R version 3.6.2 (R Core Team, 2020), considering a significant *α* ⩽0.05. A species accumulation curve was performed using the vegan package (Oksanen *et al*., 2019), in order to assess sample size adequacy, considering the presence of helminth species in each host specimen analysed.

#### Network analysis and the influence of species traits

**Fig. 1.**
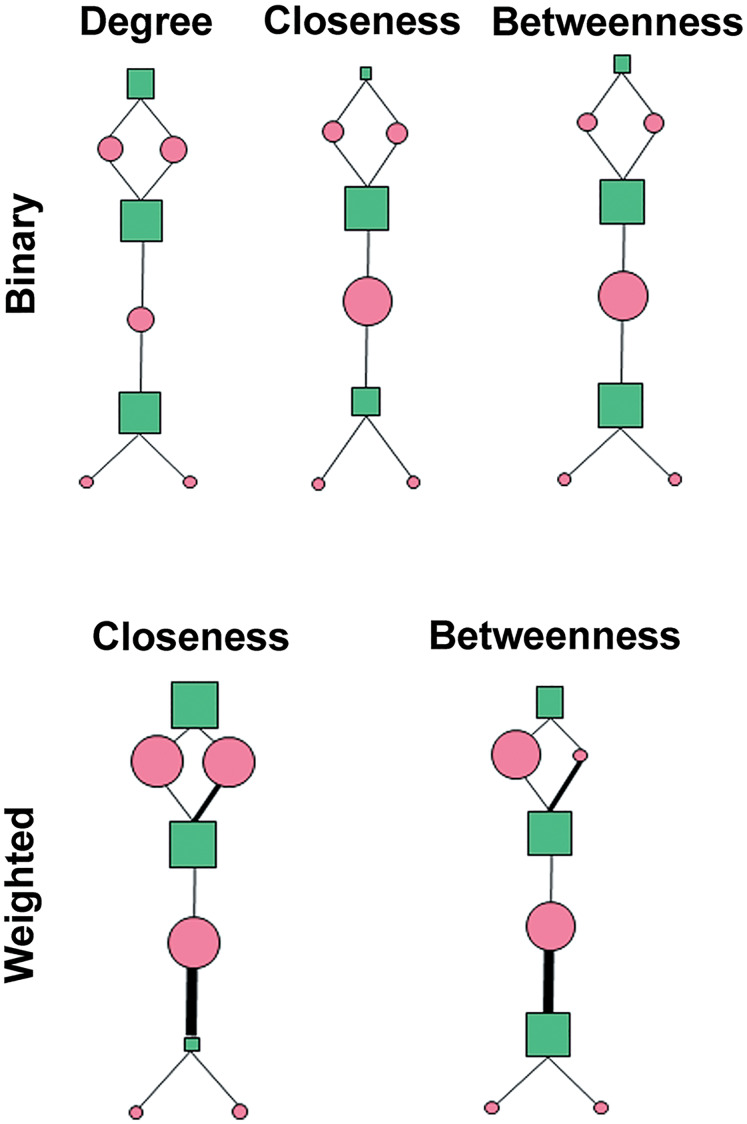
Conceptual representation of interaction networks between parasites (circles) and their hosts (squares), and species centrality (Degree, Closeness and Betweenness), using binary data of parasite occurrence in hosts and weighted data by abundance or prevalence of parasites in hosts. Degree centrality refers to the number of a node's direct connections to other nodes in the network. Betweenness centrality refers to the number of times a node lies on the shortest path between all other nodes. Closeness centrality is the average length of the shortest path connecting the node and all other nodes in the network. The thickness of the links between nodes represents the weight of interactions, considering either binary (equal weights) or weighted networks. The size of the nodes represents the values of species centralities considering binary or weighted networks.

Three node centrality statistics (degree, betweenness and closeness) were calculated to infer species roles and to estimate their importance in the structure of the host–parasite network (Newman, 2010; Dallas *et al*., 2019) (Fig. 1). Degree centrality refers to the number of a node's direct connections to other nodes in the network (Newman, 2010). This centrality metric measures the importance of species as focal points of spreading in the network. Betweenness centrality refers to the number of times a node lies on the shortest path between all other nodes, measuring how much a species intermediates the connection between all other species. Closeness centrality is the average length of the shortest path connecting the node and all other nodes in the network and it measures how close a species is to all other species in the network (Dallas *et al*., 2019). Node centrality analysis was performed for both small mammal and helminth species considering the three different parameters. In addition, network modularity (metric Q, Newman, 2006) was calculated by applying the multi-level modularity optimization algorithm (Blondel *et al*., 2008). This analysis took into account the infected animals only. Network modularity ranges from −1 (when the network is not modular) to 1 (when the network is strongly modular) and measures the density of links inside groups or modules as compared to links between modules (Blondel *et al*., 2008). Species degree, closeness and betweenness centrality metrics, and the network modularity were calculated using the igraph package (Csardi and Nepusz, 2006).

The vulnerability of host species to parasites and the dependence of parasite species on hosts were analysed using the species strength measure (SS) (Bascompte *et al*., 2006), considering the helminths mean abundance and prevalence matrices. This analysis was performed using the bipartite package (Dormann *et al*., 2008).

Associations among species centrality metrics (degree, betweenness and closeness) and species strength measure (SS) with species functional traits were investigated by Multiple Regression analysis using the vegan package (Oksanen *et al*., 2019). These analyses were carried out to investigate whether functional traits shaped species roles in the host–parasite network. We also investigated the influence of species abundances on their centrality metrics. Thus, normalized abundances of small mammals (hereafter referred to as small mammal abundance) were calculated as the total number of collected individuals divided by the abundance of the most abundant species. For the helminths, mean abundance across host species (hereafter referred to as total mean abundance) was calculated to represent the abundance of each helminth species (Supplementary Table S3).

#### Ecological and evolutionary similarities

The way that parasite and host ecological and evolutionary similarities affected their interaction patterns was tested by Multiple Regression on Distance Matrices (Lichstein, 2007), using the ecodist package (Goslee and Urban, 2007). Thus, the extent to which functionally or taxonomically similar hosts shared more parasites than dissimilar ones was investigated, as well as the extent to which parasites with greater functional and taxonomic similarity co-infected more similar hosts. For this, several distance matrices were calculated using the vegan package (Oksanen *et al*., 2019). The first matrix was built from presence and absence data of helminth species in each host species using the Jaccard qualitative index. The two other matrices were built from mean abundance and prevalence data using the Bray-Curtis quantitative index. Distance matrices of species functional traits were calculated using the Gower Dissimilarity (Gower, 1971). Taxonomic distance matrices were built using the taxa2dist function (vegan package), which generates mean taxonomic distance values for all possible pairs of species in the network.

## Results

### Parasite community structure and network patterns

**Fig. 2.**
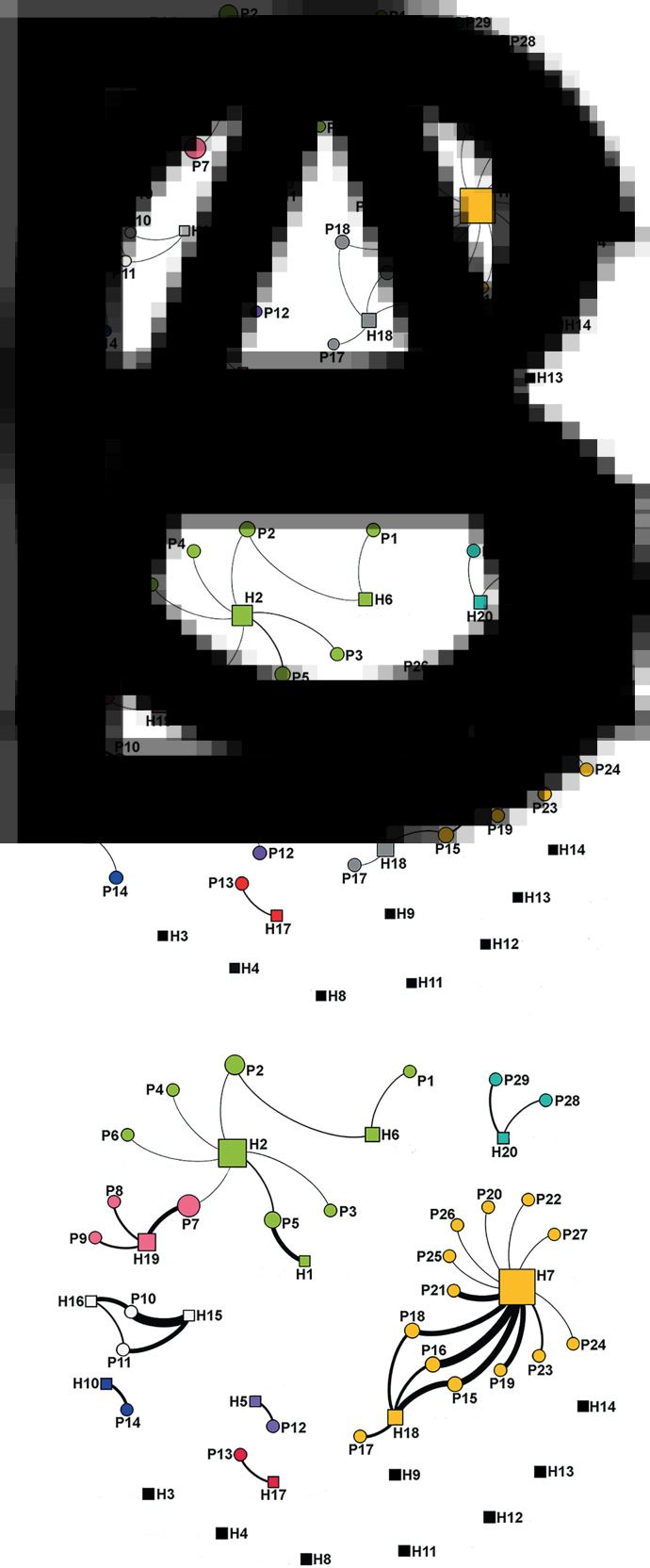
Host–parasite networks of the interactions between small mammals (square) and helminth parasites (circles), using presence and absence (A), mean abundance (B) and prevalence (C) of parasite species in each host species at Serra dos Órgãos National Park, state of Rio de Janeiro, Brazil. Small mammals represented by the black squares did not show interactions with parasites. Differences in node sizes represent different values of betweenness centrality. Thickness of the links between nodes represents different values of mean abundance (B) and prevalence of parasites (C) in their hosts. Colours represent different modules. Host species: H1. *Abrawayaomys ruschii*; H2. *Akodon montensis*; H3. *Bibimys labiosus*; H4. *Castoria angustidens*; H5. *Blarinomys breviceps*; H6. *Delomys dorsalis*; H7. *Didelphis aurita*; H8. *Euryoryzomys russatus*; H9. *Juliomys pictipes*; H10. *Marmosops incanus*; H11. *Marmosops paulensis*; H12. *Monodelphis americana*; H13. *Monodelphis iheringi;* H14. *Monodelphis scalops*; H15. *Oligoryzomys flavescens*; H16. *Oligoryzomys nigripes*; H17. *Oxymycterus quaestor*; H18. *Philander quica*; H19. *Thaptomys nigrita*; and H20. *Trinomys dimidiatus*. Helminth species: P1. *Alippistrongylus* sp.; P2. *Rodentolepis akodontis*; P3. *Stilestrongylus eta*; P4. *Trichofreitasia lenti*; P5. *Stilestrongylus aculeata*; P6. *Canaania obesa*; P7. *Protospirura numidica criceticola*; P8. *Stilestrongylus* sp.; P9. *Pterygodermatites* sp.; P10. *Stilestrongylus lanfrediae*; P11. *Guerrerostrongylus zetta*; P12. Cestoda 1; P13. *Litomosoides* sp.; P14. Cestoda 2; P15. *Aspidodera raillieti*; P16. *Cruzia tentaculata*; P17. *Viannaia* sp.; P18. *Turgida turgida*; P19. *Heterostrongylus heterostrongylus*; P20. *Mathevotaenia* sp.; P21. *Viannaia hamata*; P22. *Travassostrongylus orloffi*; P23. *Rhopalias coronatus*; P24. *Globocephalus marsupialis*; P25. *Trichuris minuta*; P26. *Trichuris didelphis*; P27. *Oligacanthorhynchus microcephalus*; P28. *Trichuris* sp.; and P29. *Heligmostrongylus* sp.

Twenty species of small mammals were captured, 12 of which were infected by at least one helminth species, including eight sigmodontine rodents, one echimid rodent and three marsupials (Fig. 2). Twenty-nine morphospecies of gastrointestinal helminths were recovered from these hosts, 22 nematodes, four cestodes, two trematodes and one acanthocephalan (Fig. 2). The species accumulation curve for helminth species richness stabilized after 73 host specimens sampled, indicating sample size adequacy (Supplementary Fig. S1). Network connectance (*C*), i.e. the proportion of realized interactions in the network, was *C* = 0.11.

The network presented modular structures for the three parameters: presence and absence (*Q* = 0.71; Fig. 2A), mean abundance (*Q* = 0.49; Fig. 2B) and prevalence (*Q* = 0.67; Fig. 2C). The degree centrality was the highest for the marsupial *D. aurita* (12) and the rodent *Akodon montensis* Thomas, 1913 (6), indicating that these two host species interacted with a higher number of helminth species in the host–parasite network (Fig. 2 and Supplementary Table S4). *Didelphis aurita* and *A. montensis* also presented the highest betweenness centrality values, regardless of the parameter used in the analysis (presence and absence – Fig. 2A, mean abundance – Fig. 2B and prevalence – Fig. 2C matrices; Supplementary Table S4). In addition, closeness centrality values did not show high variation among host species, with mean value and standard deviation equal to 4.53 × 10^−4^ ± 3.91 × 10^−4^ for the presence and absence matrix, 4.37 × 10^−4^ ± 3.73 × 10^−4^ for the mean abundance matrix and 3.44 × 10^−4^ ± 2.93 × 10^−4^ for the prevalence matrix (Supplementary Table S4).

The helminth species interacted with a maximum of two host species, presenting a degree centrality of 2 for the nematodes *Aspidodera raillieti* Travassos, 1913, *Cruzia tentaculata* (Rudolphi, 1819), *Guerrerostrongylus zetta* (Travassos, 1937) Sutton and Durette-Desset, 1991, *Protospirura numidica criceticola* (Quentin, Karimi and Rodrigues de Almeida, 1968), *Stilestrongylus aculeata* (Travassos, 1918), *Stilestrongylus lanfrediae* Souza, Digiani, Simões, Luque, Rodrigues-Silva and Maldonado Jr., 2009 and *Turgida turgida* (Rudolphi, 1819) Travassos, 1919, and for the cestode *Rodentolepis akodontis* (Rêgo,^1967^) (Fig. 2 and Supplementary Table S5). All the other parasite species occurred in only one host species (degree centrality equal to 1) (Fig. 2 and Supplementary Table S5). *Protospirura n. criceticola*, *R. akodontis*, *S. aculeata* and *T. turgida* presented high values of betweenness centrality regardless of the parameter used in the analysis (Fig. 2 and Supplementary Table S5). Similarly to the results observed for the hosts, closeness centrality also did not show high variation among helminth species with mean values and standard deviation equal to 8.25 × 10^−4^ ± 1.11 × 10^−4^ for the presence–absence matrix, 7.51 × 10^−4^ ± 1.04 × 10^−4^ for the mean abundance matrix and 5.43 × 10^−4^ ± 8.62 × 10^−5^ for the prevalence matrix (Supplementary Table S5).

**Fig. 3.**
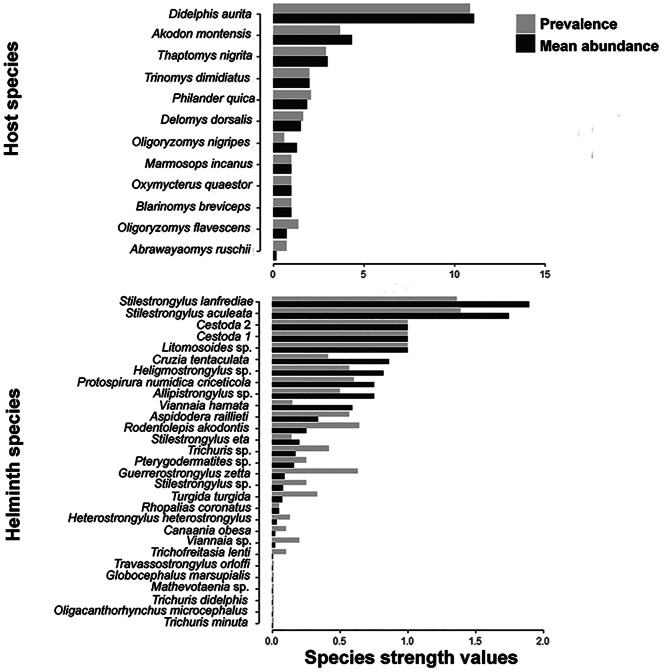
Values of species strength (SS) of host–parasite interactions considering mean abundance (black bars) and prevalence (grey bars) of each parasite species per host at Serra dos Órgãos National Park, state of Rio de Janeiro, Brazil. Values of species strength (SS) indicate the host vulnerability to infection and the parasite dependence on its hosts and are provided only for the infected small mammal species.

Considering mean abundance and prevalence parameter matrices, *D. aurita* and *A. montensis* were the hosts with the highest species strength (SS) values (Fig. 3 and Supplementary Table S6), showing the greatest vulnerability to parasite infection. In addition, *S. aculeata* and *S. lanfrediae* were the helminths with the highest SS (Fig. 3 and Supplementary Table S6), showing the highest dependence on their hosts.

### Predictors of the species interactions

Host species degree and betweenness centralities were associated with their abundances and with a frugivorous/omnivorous diet, considering the three parameters analysed (*P* < 0.01 in all cases; Table 1). However, host functional traits and abundance did not affect their closeness centrality (Table 1). For parasites, degree centrality was not influenced by their traits or abundance (Table 1). Parasite betweenness was positively influenced by the site of infection (stomach), considering the three parameters analysed. However, considering the presence–absence matrix, parasite betweenness was negatively affected by the site of infection (lungs) and parasite body length (Table 1). Yet, parasite body length showed a very small magnitude effect in the regression analysis (Table 1). In turn, helminth closeness was negatively associated with total mean abundance only when using the parasite abundance, although also with a very small magnitude effect (Table 1).

**Table 1.** Results of the multiple regression analysis relating abundances and functional traits of hosts and parasites (hosts: body length, diet, locomotor habits and activity period; parasites: body length, site of infection and type of life cycle) to the centrality metrics (degree, betweenness and closeness) and species strength (SS), considering presence and absence, mean abundance and prevalence of each parasite species per host at Serra dos Órgãos National Park, state of Rio de Janeiro, Brazil

Parameters	*R*^2^	*F*	d.f.	*P*-value of models	Trait	Estimate	*P*-value of traits
Centrality metrics							
Host betweenness (presence–absence)	0.82	11.15	9	<0.01	Host abundance	35.21	<0.01
					Frugivorous/omnivorous diet	79.16	<0.01
Host betweenness (mean abundance)	0.81	10.14	9	<0.01	Host abundance	33.77	<0.01
					Frugivorous/omnivorous diet	65.58	<0.01
Host betweenness (prevalence)	0.83	11.21	9	<0.01	Host abundance	35.39	<0.01
					Frugivorous/omnivorous diet	75.94	<0.01
Host closeness (presence–absence)	0.12	1.29	9	0.34	–	–	–
Host closeness (mean abundance)	0.06	1.13	9	0.42	–	–	–
Host closeness (prevalence)	0.10	1.22	9	0.38	–	–	–
Host degree (presence–absence)	0.84	12.31	9	<0.01	Host abundance	4.05	<0.01
					Frugivorous/omnivorous diet	7.60	<0.01
							
Parasite betweenness (presence–absence)	0.61	5.76	8	<0.01	Body length	−0.52	<0.01
					Stomach niche	23.81	<0.01
					Lungs niche	−16.85	0.05
Parasite betweenness (mean abundance)	0.89	25.07	8	<0.01	Stomach niche	29.40	<0.01
Parasite betweenness (prevalence)	0.83	16.11	8	<0.01	Stomach niche	26.14	<0.01
Parasite closeness (presence–absence)	−0.01	0.97	8	0.49	–	–	–
Parasite closeness (mean abundance)	0.55	4.80	8	<0.01	Parasite total mean abundance	<–0.01	<0.01
Parasite closeness (prevalence)	0.13	1.46	8	0.25	–	–	–
Parasite degree (presence–absence)	0.08	1.26	8	0.32	–	–	–
Species strength							
Host species strength (mean abundance)	0.99	261.3	8	<0.01	Frugivorous/omnivorous diet	10.63	<0.01
					Terrestrial locomotor habit	1.10	<0.01
					Host abundance	1.38	0.01
Host species strength (prevalence)	0.97	51.85	8	<0.01	Frugivorous/omnivorous diet	10.18	<0.01
							
Parasite species strength (mean abundance)	−0.07	0.81	8	0.60	–	–	–
Parasite species strength (prevalence)	−0.10	0.72	8	0.67	–	–	–

Only statistically significant functional traits are presented.

*R*^2^ = proportion of the total variance explained by the regression model (model goodness-of-fit), *F* = variation between sample means/variation within samples, d.f. = degrees of freedom, Estimate = *β* coefficient indicating the magnitude effect of each trait, and *P* value = significance value considering *α* ⩽0.05.

The analysis of species strength (SS) showed that host vulnerability to parasites was associated with their frugivorous/omnivorous diet (*P* < 0.01), terrestrial locomotor habit (*P* < 0.01) and abundance (*P* = 0.01), when using the parasite abundance (Table 1). In addition, an influence of the frugivorous/omnivorous diet (*P* < 0.01; Table 1) was observed on host vulnerability when considering the parasite prevalence. Functional traits, however, did not influence the dependence of parasites on their hosts (Table 1).

### Ecological and evolutionary similarities

Small mammal species with more similar functional traits, regardless of their taxonomic similarity, shared more parasite species (presence–absence: *P* = 0.03, mean abundance: *P* = 0.03 and prevalence: *P* = 0.04; Table 2). For helminths, host species sharing was not related either to functional or taxonomic traits of these parasites (*P* > 0.05 in all cases; Table 2). However, it must be taken into account that these relationships had a low goodness-of-fit (low *R*^2^ values; Table 2 and Supplementary Fig. S2) and that the sparse structure of the parasite–host network constraints the signal, particularly in mean abundance data (Supplementary Fig. S2B). For presence–absence and prevalence data, the signal was low but significantly higher than in randomized networks (Supplementary Fig. S2A and S2C).

**Table 2.** Multiple regression coefficients and goodness-of-fit of species interaction distance matrices considering presence and absence, mean abundance and prevalence of each parasite species per host, with their functional trait distance matrices and taxonomic distance matrices, at Serra dos Órgãos National Park, state of Rio de Janeiro, Brazil

Parameters	Functional traits distance matrices	Taxonomic distance matrices	*R*^2^
Host–host interaction (presence–absence)	0.03*	0.99	0.07
Host–host interaction (mean abundance)	0.03*	0.90	0.06
Host–host interaction (prevalence)	0.04*	0.95	0.06
Parasite–parasite interaction (presence–absence)	0.10	0.47	0.02
Parasite–parasite interaction (mean abundance)	0.45	0.80	<0.01
Parasite–parasite interaction (prevalence)	0.15	0.60	0.01

*P < 0.05.

## Discussion

The roles played by parasites and hosts in the network were related to their functional traits, particularly their importance in intermediating host species interactions (betweenness centrality). Parasites whose infection site was the stomach played a central role, connecting the modules of this small mammal-helminth network. Considering host species, only the frugivorous/omnivorous diet and a high abundance influenced their number of interactions (degree) and their importance in intermediating interactions among parasites (betweenness). The frugivorous/omnivorous diet, the terrestrial locomotor habit and a higher host abundance influenced the vulnerability of these animals to parasites in the environment. In addition, functionally similar host species shared more parasites.

### Hosts

Two host species, *A. montensis* and *D. aurita*, were the ones with the greatest importance in this network, hosting a great diversity of parasites, intermediating more species interactions than other hosts, and presenting the shortest distance to all other species in the network. This indicates that these hosts can quickly obtain and transmit the infection to other host species in the network. In fact, *A. montensis* shared helminths with three other host species, whereas *D. aurita* shared three helminth species with the marsupial *Philander quica* (Temminck, 1824). *Akodon montensis* and *D. aurita* are frequently found in small mammal inventories, exhibit a generalist and opportunistic behaviour (D'Andrea *et al*., 1999; Cardoso *et al*., 2016), occur in different habitats and consume a variety of food items in the environment (Carvalho *et al*., 1999; Talamoni *et al*., 2008). These ecological traits may influence their vulnerability to infection, corroborating the results of the network analysis. Previous studies have found a high number of parasite species interacting with these hosts in different environments (Püttker *et al*., 2008; Kuhnen *et al*., 2012; Cardoso *et al*., 2016; Costa-Neto *et al.*, 2019).

Central hosts (higher degree and betweenness centralities) had larger abundances and a frugivorous/omnivorous diet. Similarly, host abundance, frugivorous/omnivorous diet and terrestrial locomotor habit affected small mammals’ vulnerability to parasite infection, as indicated by the species strength measures. These results indicated the importance of small mammal population density in determining their number of interactions in the host–parasite network, as well as their sharing of parasites among different host groups. Parasites may exhibit a threshold in the host population density that is required for their successful transmission rate and local establishment (Poulin, 2007). More abundant host species may have higher parasite encounter rates in the environment than those occurring at lower densities (Kamiya *et al*., 2014; Morand, 2015; Dallas *et al*., 2019, 2020), thus increasing their number of interactions. Moreover, hosts with high population density contribute more to parasite spillover than hosts with small population sizes (Johnson *et al*., 2020). However, it must be taken into account that other factors may also affect host–parasite interactions in several spatial scales (Kamiya *et al*., 2014; Morand, 2015).

The host's diet may be considered an important factor in increasing their exposure to parasite infection (Dallas *et al*., 2019). Many helminths can be acquired by contact with infectious stages present in the environment or by the consumption of contaminated food (Leung and Koprivnikar, 2019). Host species that have an omnivorous diet can consume a variety of food items available in the environment, including fruits, vertebrates and many invertebrates, which may act as intermediate hosts. Indeed, many helminths with indirect life cycles use arthropods as intermediate hosts (Marcogliese, 2003; Poulin, 2007), such as *P. n. criceticola* and *R. akodontis*. In addition, the influence of the locomotor habit on the hosts’ vulnerability to infection may be related to the fact that terrestrial hosts may present high rates of infection by soil-transmitted helminths.

Hosts with similar functional traits tended to share more parasites among themselves. This pattern may be associated with the ecological characteristics of these organisms, as host species with greater biological and ecological similarity may exert similar selection pressures on their parasites and thus tend to share more parasite species (Poulin, 2007). Moreover, the establishment of parasites in new hosts may involve physiological pre-adaptations and ecological fitting to new conditions (Malcicka *et al*., 2015). Contrary to our expectations, however, taxonomically similar hosts did not show a greater share of parasite species among them. Due to phylogenetic conservatism, similar parasitic fauna are expected to occur among hosts that are taxonomically close (Poulin, 2014). However, in the present study, only two mammal orders were analysed, and this effect could be more evident when considering a broader range of taxonomic groups in the analysis. Therefore, phylogenetic conservatism may be scale-dependent. Indeed, Dallas *et al*. (2019) reported that phylogeny was an important predictor of the role played by host species in a network when considering several mammal orders.

Among the 20 species of small mammals captured, eight did not show helminth infections: the rodents *Bibimys labiosus* Winge, 1887, *Castoria angustidens* Winge, 1887, *Euryoryzomys russatus* Wagner, 1848 and *Juliomys pictipes* Osgood, 1933, and the marsupials *Marmosops paulensis* Tate, 1931, *Monodelphis americana* Müller, 1776, *Monodelphis iheringi* Thomas, 1888 and *Monodelphis scallops* Thomas, 1888. Most of these species were rare, which may explain the absence of interactions with parasites. In addition, parasites tend to have aggregated distribution in which few hosts are highly parasitized, exhibiting high abundance and many hosts have few or no parasites (Poulin, 2013). Therefore, the aggregated nature of the distribution of parasite–host interactions constraints the signal of the functional and taxonomic distances at the local scale.

### Parasites

The nematodes *P. n. criceticola*, *S. aculeata* and *T. turgida*, and the cestode *R. akodontis* were central helminth species, intermediating more interactions in the network (highest betweenness). These helminths, together with the nematodes *A. raillieti*, *C. tentaculata*, *G. zetta* and *S. lanfrediae*, were non-specific parasites, infecting two host species (highest degree). These parasites have already been found infecting a high diversity of host species in South America (Rêgo, 1967; Stein *et al*., 1994; Miño, 2008; Jiménez *et al.*, 2011; Simões *et al*., 2011; Panisse *et al*., 2017; Costa *et al*., 2019). Non-specific parasites shape the core of host–parasite networks because they establish multiple links among different host species (Poulin, 2010). The very small variation in closeness centrality among parasites indicated that most species were sharing very few hosts among each other. This reflects the high specificity level of the interactions, as among the 29 parasite species recovered, 21 were specific to a single host species and eight occurred in two host species. Likewise, the high modularity observed, that is, the grouping in several compartments, was a consequence of the high parasite specificity.

Concerning the parasite species dependencies on hosts in the network, the nematodes of the genus *Stilestrongylus*, *S. aculeata* and *S. lanfrediae*, which were the parasites with the highest dependence values (SS), also showed high values of mean abundance and prevalence in the rodent hosts *A. montensis* and *A. ruschii*, and in *O. flavescens* and *O. nigripes*, respectively. In this way, although these parasites were not host-specific, a high abundance and a high number of hosts infected would be necessary for the maintenance of these parasite species in the community. The genus *Stilestrongylus* is a common parasite group of sigmodontine rodents and has a strong coevolutionary history with these hosts (Simões *et al*., 2011). Sigmodontine rodents infected by *Stilestrongylus* have been widely reported by several studies in different environments (Simões *et al*., 2011; Panisse *et al*., 2017; Boullosa *et al*., 2019).

Among the parasite species that intermediated a largest number of interactions and connected modules, two of them, *T. turgida* and *P. n. criceticola*, occurred in the stomach. The occurrence in the stomach is a common pattern in the order Spirurida, of which both species belong (Anderson, 2000). In the module containing *A. montensis*, *P. n. criceticola* connected this host with the rodent *T. nigrita*, and both interacted with different exclusive parasites. In the same way, the nematode *T. turgida* intermediated the connection between the marsupials *D. aurita* and *P. quica*, and the former was the host with the highest number of exclusive parasites in the network. The negative influence of the infection niche in host lungs on parasite betweenness may be attributed to the occurrence of the nematode *Heterostrongylus heterostrongylus* Travassos, 1925 only in *D. aurita*, which was the only helminth typical of the cardiopulmonary system.

Functionally and taxonomically similar parasite species did not explore more similar host species. Although previous studies have shown that taxonomic distance explains the patterns of interactions among parasites and their hosts (Poulin *et al*., 2013; Krasnov *et al*., 2016), this relationship between small mammals and their helminths was not observed. Similarly to what was observed for the host species, it is possible that phylogenetic signal in species interaction networks may be scale-dependent and that further studies would benefit from exploring the scaling of phylogenetic signal in ecological networks.

In conclusion, host traits that better-explained species roles and importance in this small mammal-helminth network were the ones mostly related to the encounter filter of host–parasite interactions, as they represent components of behaviour. The frugivorous/omnivorous diet of the hosts and the terrestrial locomotor habit increased their vulnerability to parasite infection and, consequently, influenced species centralities. The parasite centrality was mainly explained by the infection site, which is related to the resource parameter of the compatibility filter. Thus, functional traits were important predictors of species roles in this parasite–host network and this relationship is consistent with the niche theory. Moreover, host abundance also influenced their centralities, but with a weak effect, as the more abundant the host, the stronger their interactions with parasites, which is expected by the neutral theory. These results indicate that both neutral factors and niche selection may be driving host–parasite interactions in this network. Finally, the lack of taxonomic effect on species interaction patterns opens new questions regarding the scaling of phylogenetic signal in ecological networks.

## Supplementary material

For supplementary material accompanying this paper visit https://doi.org/10.1017/S0031182021000640. click here to view supplementary material
