# Supplementary material for: Functional traits shape small mammal-helminth network: patterns and processes in species interactions
Source: Parasitology. 2021 Apr 21;148(8):947–55. doi: 10.1017/S0031182021000640 (PMC8193565; doi:10.1017/S0031182021000640)
Supplement: Supplementary file 1 [file S0031182021000640sup.zip › S0031182021000640sup001.docx]

**Table S1.** Species trait data for host and parasite species at Serra dos Órgãos National Park, state of Rio de Janeiro, Brazil.

| Trait | Description | Categories | Source |
| --- | --- | --- | --- |
| Host body length (mm) | mean body length of each host species | - | Our database |
|  |  |  |  |
| Host diet | diet of each host species | frugivorous/granivorous | Paglia *et al.* (2012) |
|  |  | frugivorous/omnivorous |  |
|  |  | frugivorous/seed predator |  |
|  |  | insectivorous/omnivorous |  |
| Host locomotor habit | locomotor habit of each host species | arboreal | Paglia *et al.* (2012) |
|  |  | scansorial |  |
|  |  | terrestrial |  |
|  |  | semi-fossorial |  |
| Host activity period | activity period of each host species | diurnal | Wilman *et al.* (2014) |
|  |  | crepuscular |  |
|  |  | nocturnal |  |
|  |  |  |  |
|  |  |  |  |
| Parasite body length (mm) | mean body length of each parasite species | - | Our database, Babero (1960) Gomes and Vicente (1972), Vicente *et al.* (1997), Digiani and Kinsella (2014) and Costa-Neto *et al.* (2019). |
| Site of infection | infection site (infection niche) of the parasite in its host | lungs | Our database |
|  |  | bile ducts |  |
|  |  | stomach |  |
|  |  | small intestine |  |
|  |  | large intestine |  |
|  |  | abdominal cavity |  |
|  |  |  |  |
| Parasite life cycle | the life cycle of each parasite species | direct | Vicente *et al*. (1997) and Anderson (2000) |
|  |  | indirect |  |

**Table S2.** Prevalence (with 95% confidence interval) and mean abundance with standard deviation of each parasite species at Serra dos Órgãos National Park, state of Rio de Janeiro, Brazil.

| Host species and helminth species | | Prevalence (confidence interval) | Mean abundance ± standard deviation |
| --- | --- | --- | --- |
| *Abrawayaomys ruschii* |  |  |  |
|  | *Stilestrongylus aculeata* | 50 (40.59-59.40) | 1.50±2.12 |
| *Akodon montensis* |  |  |  |
|  | *Canaania obesa* | 4.8 (3.71-5.96) | 0.25±1.41 |
|  | *Protospirura numidica criceticola* | 4.8 (1-13.5) | 0.06±0.30 |
|  | *Rodentolepis akodontis* | 6.5 (1.8-15.7) | 0.11±0.48 |
|  | *Stilestrongylus aculeata* | 17.7 (9.2-29.5) | 8.27±33.97 |
|  | *Stilestrongylus eta* | 6.45 (0-17.98) | 2.19±14.47 |
|  | *Trichofreitasia lenti* | 4.8 (4.66-5.01) | 0.05±0.22 |
| *Blarinomys breviceps* |  |  |  |
|  | Cestoda 1 | 25 (23.43-26.56) | 0.25±0.50 |
| *Delomys dorsalis* |  |  |  |
|  | *Alippistrongylus* sp. | 12.5 (10.1-14.8) | 0.37±1.06 |
|  | *Rodentolepis akodontis* | 12.5 (11.7-13.2) | 0.12±0.35 |
| *Oligoryzomys flavescens* |  |  |  |
|  | *Guerrerostrongylus zetta* | 50 (46.8-53.1) | 0.50±0.57 |
|  | *Stilestrongylus lanfrediae* | 100 (71-128.2) | 13.50±6.36 |
| *Oligoryzomys nigripes* |  |  |  |
|  | *Guerrerostrongylus zetta* | 20.9 (17.3-24.5) | 1.16±3.77 |
|  | *Stilestrongylus lanfrediae* | 48.8 (15.1-82.5) | 19.41±35.21 |
| *Oxymycterus quaestor* |  |  |  |
|  | *Litomosoides* sp. | 25 (0-87.7) | 10±20 |
| *Thaptomys nigrita* |  |  |  |
|  | *Protospirura numidica criceticola* | 50 (39.6-60.3) | 2.25±3.30 |
|  | *Pterygodermatites* sp. | 25 (21.8-28.1) | 0.50±10 |
|  |  |  |  |
|  | *Stilestrongylus* sp. | 25 (23.43-26.56) | 0.25±0.50 |
|  |  |  |  |
|  |  |  |  |
| *Trinomys dimidiatus* |  |  |  |
|  | *Heligmostrongylus* sp. | 23.5 (17.2-29.8) | 1.41±4.15 |
|  | *Trichuris* sp. | 17.6 (16.47-18.82) | 0.29±0.77 |
| *Didelphis aurita* |  |  |  |
|  | *Aspidodera raillieti* | 75 (27.16-122.83) | 18.58±26.42 |
|  | *Cruzia tentaculata* | 91.66 (71.85-111.48) | 19.00±10.94 |
|  | *Globocephalus marsupialis* | 8.33 (5.19-11.46) | 0.50±1.73 |
|  | *Heterostrongylus heterostrongylus* | 58.33 (40.10-76.56) | 5.00±10.07 |
|  | *Travassostrongylus orloffi* | 8.33 (5.19-11.46) | 0.50±1.73 |
|  | *Trichuris didelphis* | 8.33 (6.24-10.42) | 0.33±1.15 |
|  | *Trichuris minuta* | 8.33 (7.81-8.85) | 0.08±0.29 |
|  | *Turgida turgida* | 58.33 (43.06-73.59) | 7.75±8.43 |
|  | *Viannaia hamata* | 66.66 (0-303.82) | 88.00±131.01 |
|  | *Rhopalias coronatus* | 25.00 (0-65.95) | 7.58±22.63 |
|  | *Matevothaenia* sp. | 8.33 (6.24-10.42) | 0.33±1.15 |
|  | *Oligacanthorhynchus microcephalus* | 8.33 (7.28-9.37) | 0.17±0.58 |
| *Marmosops incanus* |  |  |  |
|  | Cestoda 2 | 33.33 (22.88-43.78) | 1.67±2.89 |
| *Philander quica* |  |  |  |
|  | *Aspidodera raillieti* | 66.66 (46.80-86.52) | 6.16±7.75 |
|  | *Cruzia tentaculata* | 33.33 (0-155.12) | 21.16±47.57 |
|  | *Turgida turgida* | 33.33 (30.68-35.97) | 0.66±1.03 |
|  | *Viannaia* sp. | 33.33 (30.23-36.43) | 0.66±1.21 |

**Table S3.** Total mean abundance with standard deviation, total Prevalence (with 95% confidence interval), life cycle and site of infection for each parasite species at Serra dos Órgãos National Park, state of Rio de Janeiro, Brazil.

| Helminth species | Mean abundance  ± standard deviation | Prevalence  (confidence interval) | Life cycle | Site of infection |
| --- | --- | --- | --- | --- |
| Phylum Nematoda |  |  |  |  |
| *Alippistrongylus* sp. | 0.38±1.06 | 12.50 (10.14-14.85) | Direct | small intestine |
| *Aspidodera raillieti* | 14.40±22.49 | 72.22 (38.98-105.46) | Direct | large intestine |
| *Cruzia tentaculata* | 19.72±27.28 | 72.22 (31.89-112.54) | Direct | large intestine |
| *Guerrerostrongylus zetta* | 1.13±3.69 | 22.22 (18.77-25.67) | Direct | small intestine |
| *Globocephalus marsupialis* | 0.5±1.73 | 8.33 (5.19-11.46) | Direct | small intestine |
| *Heligmostrongylus* sp. | 1.41±4.15 | 23.52 (17.21-29.84) | Direct | small intestine |
| *Heterostrongylus heterostrongylus* | 5.00±10.07 | 58.33 (40.10-76.56) | Indirect | lungs |
| *Litomosoides* sp. | 10.00±20.00 | 25 (0-87.70) | Indirect | abdominal cavity |
| *Protospirura numidica criceticola* | 0.20±0.93 | 7.58 (6.85-8.29) | Indirect | stomach |
| *Pterygodermatites* sp. | 0.50±1.00 | 25 (21.86-28.13) | Indirect | small intestine |
| *Stilestrongylus aculeata* | 8.06±33.46 | 18.75 (0-44.97) | Direct | small intestine |
| *Stilestrongylus eta* | 2.19±14.48 | 6.45 (0-17.98) | Direct | small intestine |
| *Stilestrongylus lanfrediae* | 19.16±34.44 | 51.11 (18.91-83.30) | Direct | small intestine |
| *Stilestrongylus* sp. | 0.25±0.50 | 25 (23.43-26.56) | Direct | small intestine |
| *Travassostrongylus orloffi* | 0.5±1.73 | 8.33 (5.19-11.46) | Direct | small intestine |
| *Trichofreitasia lenti* | 0.05±0.22 | 4.84 (4.66-5.01) | Direct | small intestine |
| *Trichuris didelphis* | 0.33±1.15 | 8.33 (6.24-10.42) | Direct | large intestine |
| *Trichuris minuta* | 0.08±0.29 | 8.33 (7.81-8.85) | Direct | large intestine |
| *Trichuris* sp. | 0.29±0.77 | 17.64 (16.47-18.82) | Direct | large intestine |
| *Turgida turgida* | 5.39±7.62 | 50 (38.73-61.26) | Indirect | stomach |
| *Viannaia hamata* | 88±131.01 | 66.66 (0-303.82) | Direct | small intestine |
| *Viannaia* sp. | 0.66±1.21 | 33.33 (30.23-36.43) | Direct | small intestine |
|  |  |  |  |  |
| Phylum Platyhelminthes |  |  |  |  |
| Class Cestoda |  |  |  |  |
| Cestoda 1 | 0.25±0.5 | 25 (23.43-26.56) | Indirect | small intestine |
| Cestoda 2 | 1.67±2.89 | 33.33 (22.88-43.78) | Indirect | small intestine |
| *Mathevotaenia* sp. | 0.33±1.15 | 8.33 (6.24-10.42) | Indirect | small intestine |
| *Rodentolepis akodontis* | 0.11±0.47 | 7.14 (6.79-7.49) | Indirect | small intestine |
| Class Trematoda |  |  |  |  |
| *Canaania obesa* | 0.26±1.41 | 4.83 (3.71-5.96) | Indirect | bile ducts |
| *Rhopalias coronatus* | 7.58±22.63 | 25 (0-65.95) | Indirect | small intestine |
|  |  |  |  |  |
| Phylum Acanthocephala |  |  |  |  |
| *Oligacanthorhynchus microcephalus* | 0.17±0.58 | 8.33 (7.28-9.37) | Indirect | small intestine |

**Table S4.** Host centrality metrics values (degree, betweenness and closeness) considering three different matrices consisting of presence and absence, mean abundance and prevalence of each parasite species per host at Serra dos Órgãos National Park, state of Rio de Janeiro, Brazil.

| Host species | Captured hosts (Infected hosts) | Degree |  | Betweenness | | |  | Closeness | | |
| --- | --- | --- | --- | --- | --- | --- | --- | --- | --- | --- |
|  |  |  |  | Presence-Absence | Mean abundance | Prevalence |  | Presence-Absence | Mean abundance | Prevalence |
| Rodents |  |  |  |  |  |  |  |  |  |  |
| *Abrawayaomys ruschii* | 2(1) | 1 |  | 0 | 0 | 0 |  | 8.41E-04 | 7.89E-04 | 4.47E-04 |
| *Akodon montensis* | 62(19) | 6 |  | 56 | 56 | 56 |  | 8.55E-04 | 8.49E-04 | 6.55E-04 |
| *Bibimys labiosus* | 2(0) | 0 |  | 0 | 0 | 0 |  | 0 | 0 | 0 |
| *Blarinomys breviceps* | 4(1) | 1 |  | 0 | 0 | 0 |  | 6.25E-04 | 6.25E-04 | 6.16E-04 |
| *Castoria angustidens* | 1(0) | 0 |  | 0 | 0 | 0 |  | 0 | 0 | 0 |
| *Delomys dorsalis* | 8(2) | 2 |  | 11 | 11 | 11 |  | 8.44E-04 | 8.48E-04 | 5.94E-04 |
| *Euryoryzomys russatus* | 1(0) | 0 |  | 0 | 0 | 0 |  | 0 | 0 | 0 |
| *Juliomys pictipes* | 1(0) | 0 |  | 0 | 0 | 0 |  | 0 | 0 | 0 |
| *Oligoryzomys flavescens* | 2(2) | 2 |  | 0.5 | 2 | 0 |  | 6.57E-04 | 6.52E-04 | 5.75E-04 |
| *Oligoryzomys nigripes* | 43(23) | 2 |  | 0.5 | 0 | 1 |  | 6.57E-04 | 6.51E-04 | 6.03E-04 |
| *Oxymycterus quaestor* | 4(1) | 1 |  | 0 | 0 | 0 |  | 6.25E-04 | 6.22E-04 | 6.16E-04 |
| *Thaptomys nigrita* | 4(2) | 3 |  | 21 | 21 | 21 |  | 8.47E-04 | 8.38E-04 | 5.26E-04 |
| *Trinomys dimidiatus* | 17(5) | 2 |  | 1 | 1 | 1 |  | 6.41E-04 | 6.41E-04 | 6.25E-04 |
|  |  |  |  |  |  |  |  |  |  |  |
| Marsupials |  |  |  |  |  |  |  |  |  |  |
| *Didelphis aurita* | 12(12) | 12 |  | 82.5 | 81 | 81 |  | 9.23E-04 | 8.15E-04 | 5.86E-04 |
| *Marmosops incanus* | 3(1) | 1 |  | 0 | 0 | 0 |  | 6.25E-04 | 6.25E-04 | 6.13E-04 |
| *Marmosops paulensis* | 1(0) | 0 |  | 0 | 0 | 0 |  | 0 | 0 | 0 |
| *Monodelphis americana* | 1(0) | 0 |  | 0 | 0 | 0 |  | 0 | 0 | 0 |
| *Monodelphis iheringi* | 3(0) | 0 |  | 0 | 0 | 0 |  | 0 | 0 | 0 |
| *Monodelphis scalops* | 4(0) | 0 |  | 0 | 0 | 0 |  | 0 | 0 | 0 |
| *Philander quica* | 6(4) | 4 |  | 14.5 | 26 | 16 |  | 9.10E-04 | 7.80E-04 | 4.26E-04 |

**Table S5.** Parasite centrality metrics values (degree, betweenness and closeness) considering three different matrices consisting of presence and absence, mean abundance and prevalence of each parasite species per host at Serra dos Órgãos National Park, state of Rio de Janeiro, Brazil.

| Helminth species | Degree |  | Betweenness | | |  | Closeness | | |
| --- | --- | --- | --- | --- | --- | --- | --- | --- | --- |
|  |  |  | Presence-Absence | Mean abundance | Prevalence |  | Presence-Absence | Mean abundance | Prevalence |
| Phylum Nematoda |  |  |  |  |  |  |  |  |  |
| *Alippistrongylus* sp. | 1 |  | 0 | 0 | 0 |  | 8.36E-04 | 8.45E-04 | 5.49E-04 |
| *Aspidodera raillieti* | 2 |  | 6.67 | 0 | 0 |  | 9.16E-04 | 7.35E-04 | 4.20E-04 |
| *Cruzia tentaculata* | 2 |  | 6.67 | 0 | 0 |  | 9.16E-04 | 6.89E-04 | 4.08E-04 |
| *Guerrerostrongylus zetta* | 2 |  | 0.5 | 2 | 1 |  | 6.57E-04 | 6.52E-04 | 6.03E-04 |
| *Globocephalus marsupialis* | 1 |  | 0 | 0 | 0 |  | 9.12E-04 | 8.11E-04 | 5.51E-04 |
| *Heligmostrongylus* sp. | 1 |  | 0 | 0 | 0 |  | 6.41E-04 | 6.41E-04 | 6.16E-04 |
| *Heterostrongylus heterostrongylus* | 1 |  | 0 | 0 | 0 |  | 9.12E-04 | 7.74E-04 | 4.06E-04 |
| *Litomosoides* sp. | 1 |  | 0 | 0 | 0 |  | 6.25E-04 | 6.22E-04 | 6.16E-04 |
| *Protospirura numidica criceticola* | 2 |  | 27 | 27 | 27 |  | 8.52E-04 | 8.49E-04 | 6.45E-04 |
| *Pterygodermatites* sp. | 1 |  | 0 | 0 | 0 |  | 8.39E-04 | 8.34E-04 | 4.60E-04 |
| *Stilestrongylus aculeata* | 2 |  | 11 | 11 | 11 |  | 8.49E-04 | 7.99E-04 | 5.93E-04 |
| *Stilestrongylus eta* | 1 |  | 0 | 0 | 0 |  | 8.47E-04 | 8.32E-04 | 6.26E-04 |
| *Stilestrongylus lanfrediae* | 2 |  | 0.5 | 0 | 0 |  | 6.57E-04 | 6.41E-04 | 5.76E-04 |
| *Stilestrongylus* sp. | 1 |  | 0 | 0 | 0 |  | 8.39E-04 | 8.36E-04 | 4.60E-04 |
| *Travassostrongylus orloffi* | 1 |  | 0 | 0 | 0 |  | 9.12E-04 | 8.11E-04 | 5.51E-04 |
| *Trichofreitasia lenti* | 1 |  | 0 | 0 | 0 |  | 8.47E-04 | 8.49E-04 | 6.34E-04 |
| *Trichuris didelphis* | 1 |  | 0 | 0 | 0 |  | 9.12E-04 | 8.12E-04 | 5.51E-04 |
| *Trichuris minuta* | 1 |  | 0 | 0 | 0 |  | 9.12E-04 | 8.14E-04 | 5.51E-04 |
| *Trichuris* sp. | 1 |  | 0 | 0 | 0 |  | 6.41E-04 | 6.41E-04 | 6.19E-04 |
| *Turgida turgida* | 2 |  | 6.67 | 30 | 20 |  | 9.16E-04 | 7.83E-04 | 4.73E-04 |
| *Viannaia hamata* | 1 |  | 0 | 0 | 0 |  | 9.12E-04 | 4.22E-04 | 3.88E-04 |
| *Viannaia* sp. | 1 |  | 0 | 0 | 0 |  | 8.99E-04 | 7.75E-04 | 3.59E-04 |
| Phylum Platyhelminthes |  |  |  |  |  |  |  |  |  |
| Class Cestoda |  |  |  |  |  |  |  |  |  |
| Cestoda 1 | 1 |  | 0 | 0 | 0 |  | 6.25E-04 | 6.25E-04 | 6.16E-04 |
| Cestoda 2 | 1 |  | 0 | 0 | 0 |  | 6.25E-04 | 6.25E-04 | 6.13E-04 |
| *Mathevotaenia* sp. | 1 |  | 0 | 0 | 0 |  | 9.12E-04 | 8.12E-04 | 5.51E-04 |
| *Rodentolepis akodontis* | 2 |  | 20 | 20 | 20 |  | 8.50E-04 | 8.49E-04 | 6.36E-04 |
| Class Trematoda |  |  |  |  |  |  |  |  |  |
| *Canaania obesa* | 1 |  | 0 | 0 | 0 |  | 8.47E-04 | 8.47E-04 | 6.34E-04 |
| *Rhopalias coronatus* | 1 |  | 0 | 0 | 0 |  | 9.12E-04 | 7.54E-04 | 4.92E-04 |
|  |  |  |  |  |  |  |  |  |  |
| Phylum Acanthocephala |  |  |  |  |  |  |  |  |  |
| *Oligacanthorhynchus microcephalus* | 1 |  | 0 | 0 | 0 |  | 9.12E-04 | 8.13E-04 | 5.51E-04 |

**Table S6.** Species strength measures of host-parasite interactions considering mean abundance and prevalence of each parasite species per host at Serra dos Órgãos National Park, state of Rio de Janeiro, Brazil. Only infected hosts were considered in this analysis. Species strength values are provided only for infected small mammals.

| Host species | Species strength measures abundance | Species strength measures prevalence |  | Helminth species | Species strength measures abundance | Species strength measures prevalence |
| --- | --- | --- | --- | --- | --- | --- |
| Rodents |  |  |  | Phylum Nematoda |  |  |
| *Abrawayaomys ruschii* | 0.15 | 0.73 |  | *Alippistrongylus* sp. | 0.75 | 0.50 |
| *Akodon montensis* | 4.35 | 3.69 |  | *Aspidodera raillieti* | 0.34 | 0.57 |
| *Blarinomys breviceps* | 1 | 1 |  | *Cruzia tentaculata* | 0.86 | 0.41 |
| *Delomys dorsalis* | 1.52 | 1.65 |  | *Guerrerostrongylus zetta* | 0.09 | 0.63 |
| *Oligoryzomys flavescens* | 0.71 | 1.37 |  | *Globocephalus marsupialis* | <0.01 | 0.01 |
| *Oligoryzomys nigripes* | 1.28 | 0.62 |  | *Heligmostrongylus* sp. | 0.82 | 0.57 |
| *Oxymycterus quaestor* | 1 | 1 |  | *Heterostrongylus heterostrongylus* | 0.03 | 0.13 |
| *Thaptomys nigrita* | 2.97 | 2.91 |  | *Litomosoides* sp. | 1 | 1 |
| *Trinomys dimidiatus* | 2 | 2 |  | *Protospirura numidica criceticola* | 0.75 | 0.60 |
|  |  |  |  | *Pterygodermatites* sp. | 0.16 | 0.25 |
| Marsupials |  |  |  | *Stilestrongylus aculeata* | 1.75 | 1.39 |
| *Didelphis aurita* | 11.14 | 10.89 |  | *Stilestrongylus eta* | 0.20 | 0.14 |
| *Marmosops incanus* | 1 | 1 |  | *Stilestrongylus lanfrediae* | 1.90 | 1.36 |
| *Philander quica* | 1.85 | 2.10 |  | *Stilestrongylus* sp. | 0.08 | 0.25 |
|  |  |  |  | *Travassostrongylus orloffi* | <0.01 | 0.01 |
|  |  |  |  | *Trichofreitasia lenti* | <0.01 | 0.10 |
|  |  |  |  | *Trichuris didelphis* | <0.01 | 0.01 |
|  |  |  |  | *Trichuris minuta* | <0.01 | 0.01 |
|  |  |  |  | *Trichuris* sp. | 0.17 | 0.42 |
|  |  |  |  | *Turgida turgida* | 0.07 | 0.33 |
|  |  |  |  | *Viannaia hamata* | 0.59 | 0.15 |
|  |  |  |  | *Viannaia* sp. | 0.02 | 0.20 |
|  |  |  |  |  |  |  |
|  |  |  |  | Phylum Platyhelminthes |  |  |
|  |  |  |  | Class Cestoda |  |  |
|  |  |  |  | Cestoda 1 | 1 | 1 |
|  |  |  |  | Cestoda 2 | 1 | 1 |
|  |  |  |  | *Mathevotaenia* sp. | <0.01 | 0.01 |
|  |  |  |  | *Rodentolepis akodontis* | 0.25 | 0.64 |
|  |  |  |  | Class Trematoda |  |  |
|  |  |  |  | *Canaania obesa* | 0.02 | 0.10 |
|  |  |  |  | *Rhopalias coronatus* | 0.05 | 0.05 |
|  |  |  |  |  |  |  |
|  |  |  |  | Phylum Acanthocephala |  |  |
|  |  |  |  | *Oligacanthorhynchus microcephalus* | <0.01 | 0.01 |

**Supplementary figure legend**

**Fig. S1.** Helminth species accumulation curve considering each infracommunity (host specimen) at Serra dos Órgãos National Park, state of Rio de Janeiro, Brazil.

**Fig. S2.** Null distribution of R-squared values from the analysis of ecological and evolutionary similarities between hosts considering the parasite population parameters: (A) presence and absence of observed interactions, (B) mean abundance of each parasite per host species and (C) prevalence of each parasite per host species. The red line indicates the observed R-squared value.

**Supplementary References**

**Anderson, RC** (2000) *Nematode Parasites of Vertebrates. Their Development and Transmission*, 2nd Edn. Farnham Royal, UK: CAB International.

**Babero, BB** (1960) Further studies on helminths of the opossum, *Didelphis virginiana*, with a description of a new species from this host. *The Journal of Parasitology* **46**, 455–463. doi: 10.2307/3275138.

**Costa-Neto, SF, Cardoso, TS, Boullosa, RG, Maldonado, A and Gentile, R** (2019) Metacommunity structure of the helminths of the black-eared opossum *Didelphis aurita* in peri-urban, sylvatic and rural environments in south-eastern Brazil. *Journal of Helminthology* **93**, 720–731. doi: 10.1017/S0022149X18000780.

**Digiani, MC and Kinsella, JM** (2014) A new genus and species of Heligmonellidae (Nematoda: Trichostrongylina) parasitic in *Delomys dorsalis* (Rodentia: Sigmodontinae) from Misiones, Argentina. *Folia parasitologica* **61**, 473–478.

**Gomes, DC and Vicente, JJ** (1972) Estudo do gênero *Rhopalias* Stiles & Hassall, 1898 (Trematoda, Rhopaliasidae). *Memórias do Instituto Oswaldo Cruz* **70**, 115–133. doi: 10.1590/S0074-02761972000200001.

**Paglia, AP, Fonseca, GAB, Rylands, AB, Herrmann, G, Aguiar, LMS, Chiarello, AG, Leite, YLR, Costa, LP, Siciliano, S, Kierulff, MCM, Mendes, SL, Mittermeier, RA and Patton, JL** (2012) Annotated checklist of Brazilian mammals 2° Edição. *Occasional Papers in Conservation Biology* **6,** 1-76.

**Vicente, JJ, Rodrigues, HO, Gomes, DC and Pinto, RM** (1997) Nematóides do Brasil. Parte V: nematóides de mamíferos. *Revista Brasileira de Zoologia* **14**, 1–452. doi: 10.1590/S0101-81751997000500001.

**Wilman, H, Belmaker, J, Simpson, J, de la Rosa, C, Rivadeneira, MM and Jetz, W** (2014) EltonTraits 1.0: Species-level foraging attributes of the world’s birds and mammals. *Ecology* **95**, 2027–2027. doi: 10.1890/13-1917.1.
