# Supplementary figures and images for: Functional traits shape small mammal-helminth network: patterns and processes in species interactions
Source: Parasitology. 2021 Apr 21;148(8):947–55. doi: 10.1017/S0031182021000640 (PMC8193565; doi:10.1017/S0031182021000640)

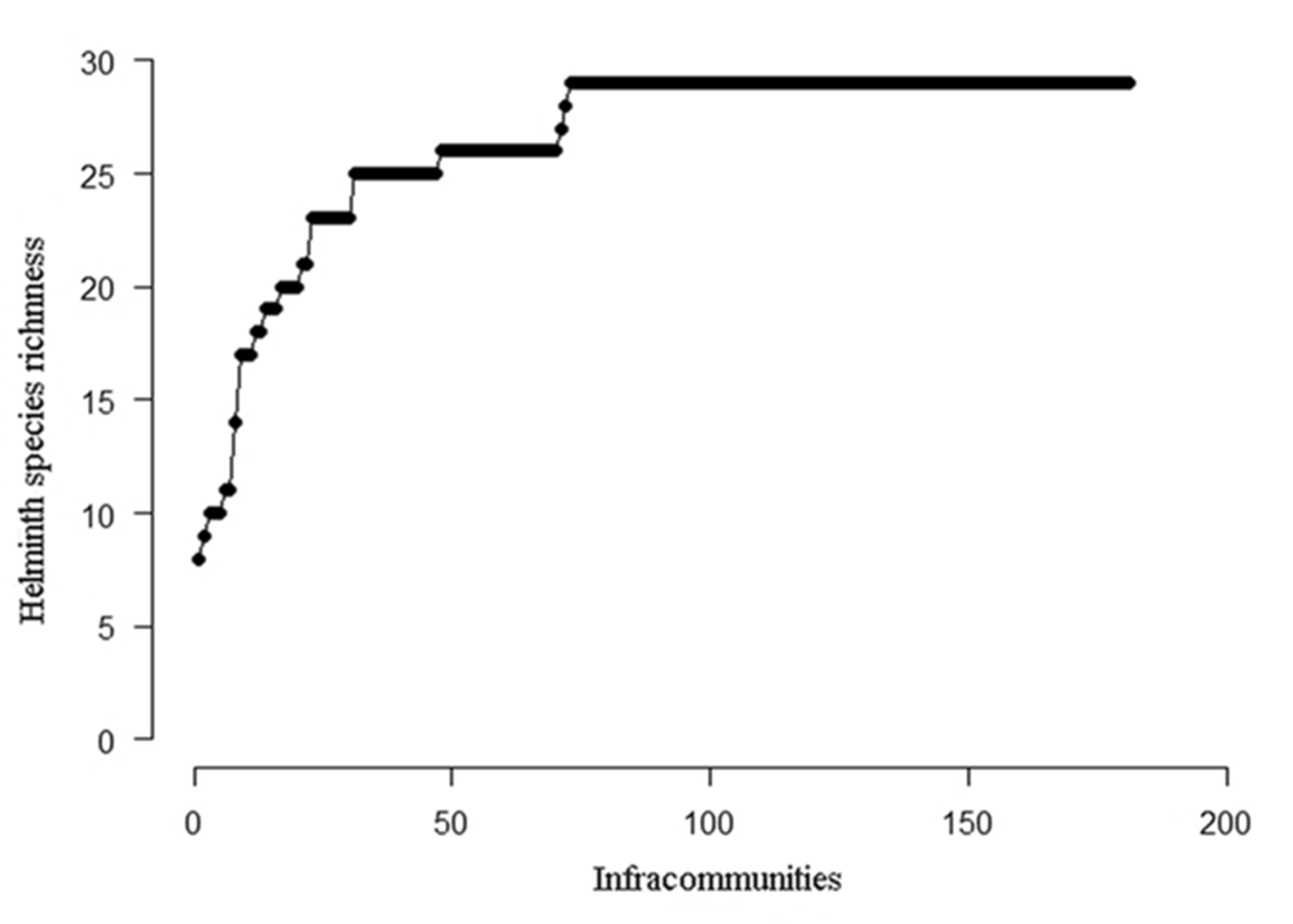

Supplement: Supplementary file 1 [file S0031182021000640sup.zip › S0031182021000640sup002.tif]

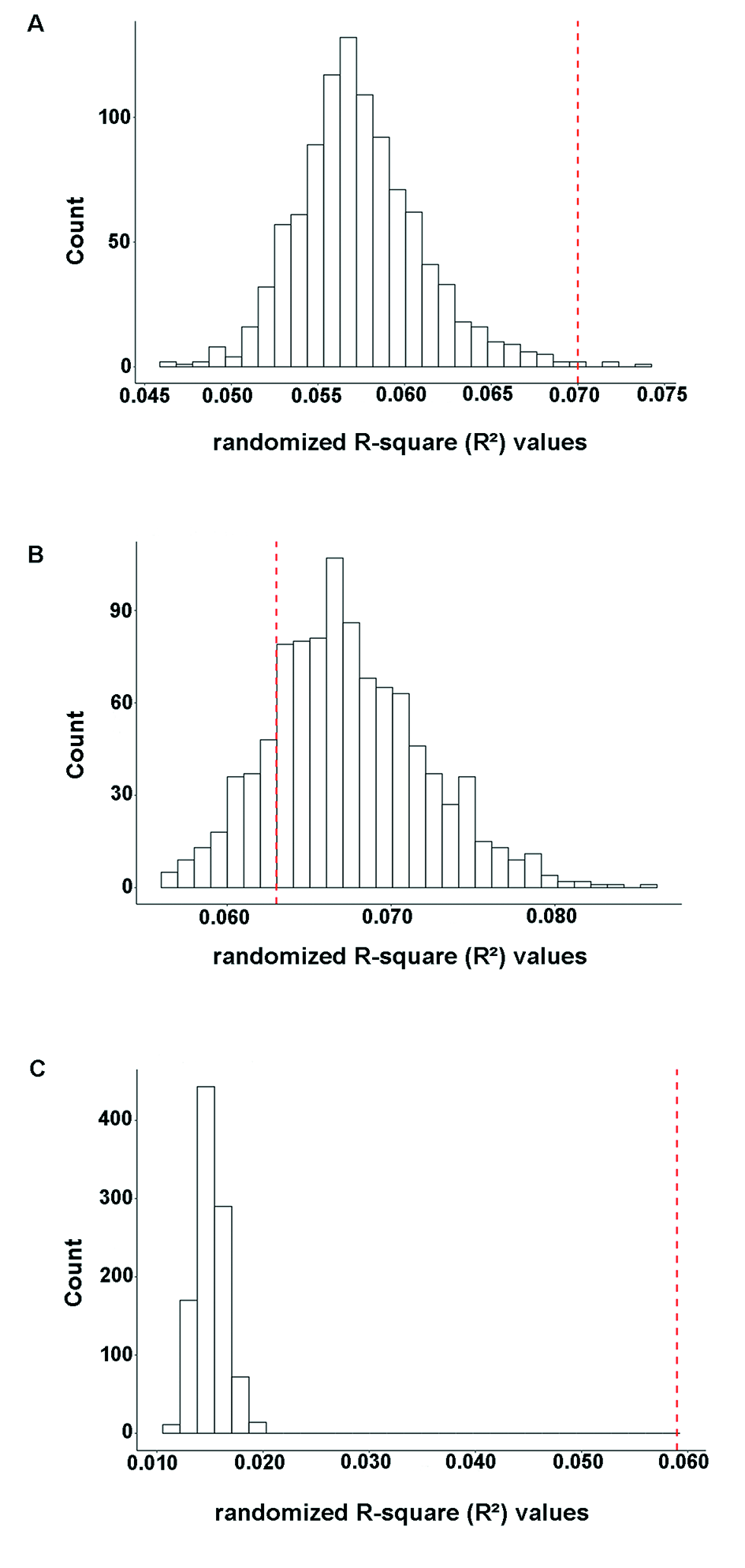

Supplement: Supplementary file 1 [file S0031182021000640sup.zip › S0031182021000640sup003.tif]
